# Preparation of an Antimicrobial and Antioxidant Bio-Polymer Film and Its Application as Glazing Shell for Postharvest Quality of Fresh-Cut Apple

**DOI:** 10.3390/foods11070985

**Published:** 2022-03-28

**Authors:** Zhaohui Yang, Yalan Zhang, Yihui Zhao, Hao Dong, Jian Peng, Qi He

**Affiliations:** 1 School of Public Health, Southern Medical University, Guangzhou 510515, China; zhaohui163@smu.edu.cn (Z.Y.); zhangyalan@i.smu.edu.cn (Y.Z.); zyhy_0089@i.smu.edu.cn (Y.Z.); 2 Research Center for Green Development of Agriculture, South China Agricultural University, Guangzhou 510642, China; 3 College of Light Industry and Food Sciences, Zhongkai University of Agriculture and Engineering, Guangzhou 510225, China; donghao@zhku.edu.cn; 4 Sericultural & Agri-Food Research Institute, Guangdong Academy of Agricultural Sciences/Key Laboratory of Functional Foods, Ministry of Agriculture and Rural Affairs/Guangdong Key Laboratory of Agricultural Products Processing, Guangzhou 510640, China; pengjian@gdaas.cn

**Keywords:** postharvest quality, fresh-cut apple, chitosan, *Zanthoxylum*

## Abstract

The aim of this work is to glazing a modified bio-polymer shell as substitute of peel to keep the postharvest quality of fresh-cut fruits. In this study, chitosan as backbone of the shell was modified by addition of the functional extracts obtained from *Zanthoxylum,* in which 12 kinds of main identified bio-active components consisted of over 55% of the total extracts. The introduction of the extracts improved physic and mechanical properties of the shell, and endowed it with significant antimicrobial and antioxidant activity. Accordingly, the modified chitosan was used as the substitute of peel to preserve fresh-cut apples. Results exhibited that such treatments obviously delayed the decline process of overall postharvest quality of the preserved apple samples throughout all the storage period, represented by the variations in physical, chemical, and microbial properties of the apple samples were significantly inhibited. The overall observations revealed promising potential of the bio-polymer shell in food application.

## 1. Introduction

Consumers always prefer their foods with improved safety, maximized edible quality and extended shelf life during the produce, transport and storage periods. Thus, postharvest preservation is often considered as a major means to maintain the freshness of fruits and vegetables and prolong their distribution cycle [1]. When it comes to fresh-cut fruits, because most of products have lost their peel as protective barrier, it calls for a more effective improvement to keep their postharvest quality during storage.

A strategy is adopted by forming a “peel substitute” using some edible bio-polymer materials. As a protective barrier on the surface of the preserved fruits, one of its major functions is to eliminate external impacts caused by contact of oxygen or microorganisms [2]. Additionally, many biopolymers have lots of functional bio-activities, thus they can also scavenge harmful components formed in food, or inhibit the variations in food properties during storage. Thus, the decline process of the postharvest quality of preserved fruits can further be slowed down [3].

Among different biopolymer that is suitable as surface protective shell, chitosan has exhibited its advantages in many applications because of its distinctive properties including antimicrobial activity, biodegradability and film-forming ability [4]. Chitosan can readily form a thin film after dissolution into a slightly acidic aqueous solution [5]. Thus, some agents are often introduced into chitosan as functional additive agents, especially some plant extracts that is easy to produce anions to help chitosan form films and improve the physical and chemical properties of the film [6,7]. Another noteworthy advantage of these composite materials is that the load of these extracts in chitosan can limit their release or decomposed and prolong their acting time, as many bio-active compositions in the extracts are labile [8,9].

In this study, *Zanthoxylum* extracts with antimicrobial and antioxidant activity were used as a functional additive agent into chitosan. Results revealed it can effectively help to form film and improve the performance of chitosan film, especially when it was used as glazing shell for fresh-cut fruits. To our knowledge, there is none or less study that has displayed such “artificial peel” made using chitosan with *Zanthoxylum* extracts and its application for fresh-cut apple. The overall findings in the study would be instructive for the real distribution chain.

## 2. Materials and Methods

### 2.1. Preparation of the Casting Solutions

Whole herbs of two *Zanthoxylum* species, i.e., *Z. acanthopodium* (ZA) and *Z. simulans* (ZS) were, respectively, collected from Hunan Province and Yunnan Province, China. Their aerial parts were dried at 50 °C for 48 h. The materials were subjected into a Clevenger-type apparatus (Kesijia Ltd., Beijing, China) for 6 h of hydrodistillation [10]. Extracts were collected and the main components were analyzed using a GC-MS system (6890-5975, Agilent Technologies, Santa Clara, CA, USA) equipped with HP-5 MS fused silica capillary column [11].

Chitosan (CAS 9012-76-4; deacetylation degree of 75~85%; medium molecular weight; viscosity: 200~800 cP) were obtained from Sigma-Aldrich Co., USA. 1 g of chitosan was dissolved in 100 mL of acetic acid (1% *v*/*v*) aqueous solution [5]. 1 mL of oleic acid was added as hydrophobic compound. 7 groups of similar solutions were prepared, namely CH, ZA1, ZA2, ZA3, ZS1, ZS2, and ZS3. 50 µL, 100 µL and 150 µL of ZA extracts were, respectively, added in the solution of ZA1, ZA2, and ZA3 groups, while equal dose of ZS extracts were added ZS1, ZS2 and ZS3. The solution with *Zanthoxylum* extracts were stirred by 400 rpm at 60 °C for 3 h and then treated by 40 KHz of ultrasound for 30 min.

### 2.2. Characterization of the Chitosan Films

The prepared solutions were cast to films using inorganic glass plates (36 cm × 24 cm) and then dried in an incubator at 60 °C for 12 h to form films. A series of assays were performed to determine the properties of the prepared films.

Micro-morphology of the films was observed using a scanning electron microscope (SEM, S-3700 N, Hitachi Corp., Osaka, Japan) and an atomic force microscope (AFM, MultiMode 8SPM, Bruker Corp., Karlsruhe, Germany) [12].

Color parameters of the films were determined using a colorimeter (CR-300, Konica Minolta, Grand Rapids, MI, USA) [13]. The color of the samples was represented by three color parameters namely L* (lightness, black = 0 and white = 100), a* (green = −a* and red = +a*), and b* (blue = −b* and yellow = +b*).

Thickness of the films was measured by a hand-held digital micrometer (Mitutoyo, Mitutoyo Corporation, Kanagawa, Japan). 20 random films were measured and the results were obtained by average.

Mechanical properties of the films were tested using a texture analyzer (TMS-Pro, Food Technology Corp., Sterling, VA, USA) [4]. Each film strip (100 mm × 6 mm) was mounted on the analyzer with initial grip distance of 50 mm and moving speed of 6 mm/min. 

Porosity properties of the films were obtained by a Brunauer-Emmett-Teller (BET) surface area analyzer (JW-BK222, JWGB, Beijing, China) [12]. The distribution of pore size was obtained from the equipped analysis software through the N_2_ adsorption and desorption curve.

Water vapor permeability (WVP) of the films was determined using a self-designed measured cup with distilled water at ~20 °C for 48 h [14]. WVP was calculated using the weight loss in the cup at intervals of 4 h.

### 2.3. Antioxidant and Antimicrobial Activity of the Chitosan Films

Antioxidant activity of the films was analyzed through the radical scavenging capacity of 2, 2-diphenyl-1-picrylhydrazyl (DPPH) and 2, 2-azinobis (3-ethylbenzothiazoline-6-sulfonic) acid radical cation (ABTS^+^) [15]. DPPH radical solution was prepared using 0.1 mmol/L of DPPH-methanolic solution. ABTS^+^ radical solution was prepared using the mixture with equal volume of ABTS^+^ (2 mmol/L) and potassium persulfate (2.45 mmol/L). The measured liquid of each material was the supernatant prepared by mixing 0.2 g of material into 3.0 mL of methanol with homogenization and centrifugation. 3 mL of measured liquid was mixed with 1 mL of radical solution. The results were calculated using the absorbances at 517 nm (DPPH) and 734 nm (ABTS^+^) obtained by a spectrophotometer (UV-1800, Shimadzu, Kyoto, Japan). *Zanthoxylum* extracts were used as positive control agents.

Antimicrobial activity of the films was observed from the inhibition zones of three microbial strains namely *Staphylococcus aureus*, *Pseudomonas aeruginosa* and *Escherichia coli*. [4]. The diameter of the zones was measured by placing a film disc (6 mm of diameter) on inoculated plates after incubation at 37 °C for 24 h.

### 2.4. The Storage of the Apples with the Glazing Shell

Apple (*Malus domestica var. Anna*) fruits (233 g ± 17 g, ~75% maturities, full color, without visible defect or decay) were collected from the Wang’s Farm, Guangzhou city, China. They were cut into cubes (~2 cm) after peeled and cored. The cubes were sanitized by dipping into 0.02% of NaClO solution. After 2 min, the cubes were washed thoroughly to avoid the disturbance of Cl. 

Subsequently, the processed cubes were divided into eight groups. The control samples (C) were directly stored without further coating treatment, while samples in CH, ZA1, ZA2, ZA3, ZS1, ZS2, and ZS3 groups were, respectively, glazed a shell using corresponding casting solutions. All samples were stored at 1 ± 0.3 °C for 15 d. With a 3 days’ interval, some samples were taken out and evaluated. 

### 2.5. Postharvest Quality Analysis of the Preserved Apples

A series of assays were performed to determine the postharvest quality of the apple samples. Sensory qualities of the samples were evaluated by 10 trained research using a 5-point hedonic scale (0, rejected to 5, fresh extremely) for 5 items namely color, odor, texture, taste, and general acceptability [16].

Firmness of the samples was evaluated by a texture analyzer (Brookfield-CT3, Brookfield, Middleboro, MA, USA) using 5 mm/s of test speed, 5 g of trigger load and 5 mm of depth [4].

Surface color of the samples was measured using a colorimeter (CR-300, Konica Minolta, Grand Rapids, MI, USA) [17].

Microbial counts of the samples were determined through total bacterial counts (TBC) and total anaerobic counts (TAC) [13]. 5 g of minced apple samples was aseptically homogenized with 45 mL sterile physiological saline for 1 min. The homogenized sample was serially diluted using 9 mL of sterile saline for microbial analysis. The counts were, respectively, obtained on the spread plates of plate count agar after 72 h of corresponding incubation at 15 °C.

Anthocyanins content in the samples was obtained by the absorbance at 535 nm using the methods described by [18]. Chlorophyll a, b and carotenoids content in the samples were, respectively, obtained by the absorbance at 440, 644 and 662 nm [4].

### 2.6. Statistical Analysis

In each assay, repeats were performed at least 3 times on different representative samples. Data were expressed as mean ± standard deviation (SD). Statistical analysis was performed using the software of SPSS (17.0, IBM, Armonk, NY, USA).

## 3. Results and Discussion

### 3.1. The Formation of the Chitosan Films

**Figure 1:**
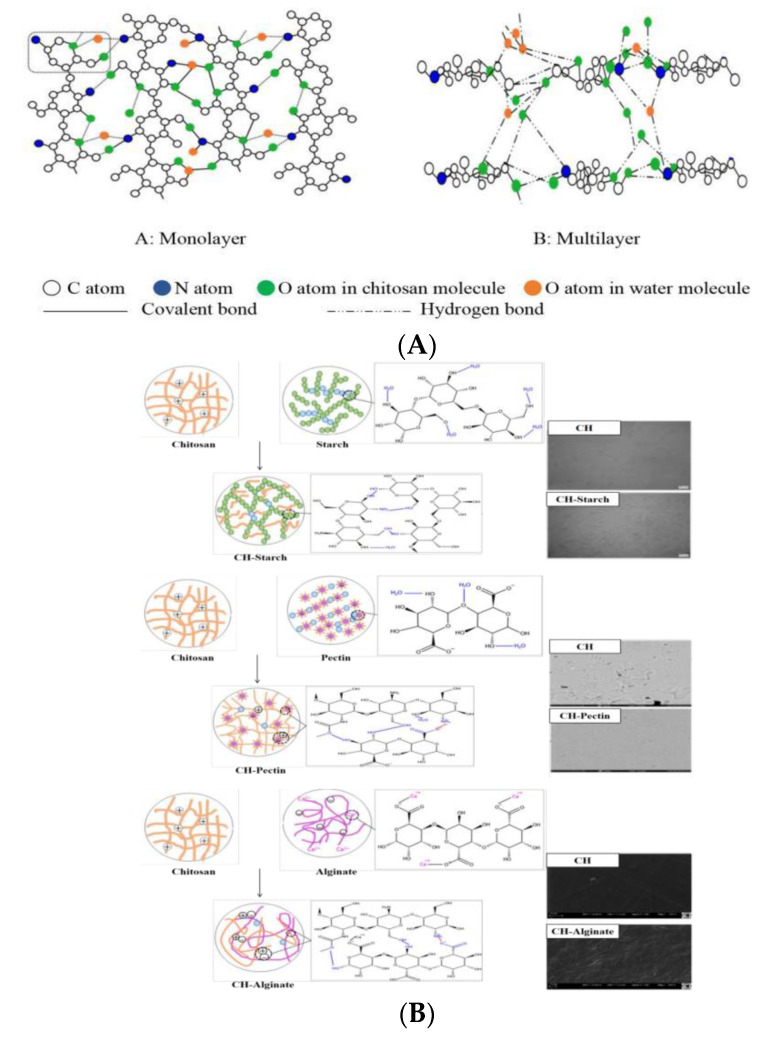

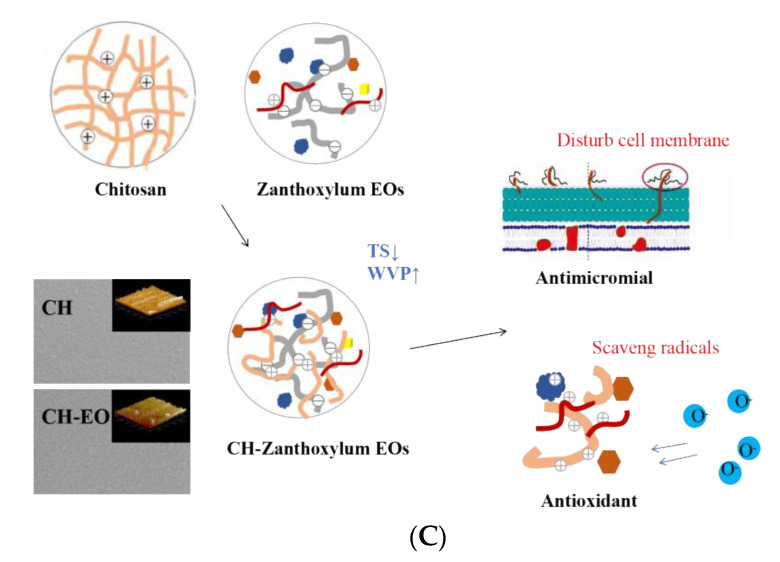
Formation mechanism of the chitosan films. (**A**) Intramolecular and intermolecular interaction of chitosan; (**B**) Different tapes of chitosan complexes; and (**C**) Chitosan-*Zanthoxylum* extracts complexes. WVP: Water vapor permeability; TS: Tensile Strength.

The formation of chitosan films under acidic conditions is revealed in Figure 1A [19]. Dissolution of chitosan in acetic solution results in intramolecular and intermolecular hydrogen bonds by connecting C_2_-NH_2_, C_3_-OH and C_6_-OH in chitosan molecule with oxygen atoms in the solution. Meanwhile, NH^3+^ groups produced from chitosan molecules would combine with anions in the solution through ionic bonds to form a three-dimensional network. Consequently, a visual phenomenon is that chitosan molecules rapidly absorb water and expand, and evenly disperse in the acidic solution. During the process of solution evaporation, hydrophobic interaction and entanglement among chitosan molecules are enhanced, and finally it forms a solid film.

The introduction of additive agents into chitosan molecules would change micro-structure and physical properties of chitosan films. As shown in Figure 1B, there is three typical categories reportedly: (1) CH-Starch type [20]. Initially, starch needs to be pretreated by gelatinization. During this process, hydrogen bonds are formed between -OH in starch molecules and -OH in water molecules. When chitosan mixed with gelatinized starch, hydrogen bonds among starch molecules, and between starch and water break down. Then they would be combined with -NH_2_ in chitosan molecules because of stronger polarity. Therefore, the starch molecules are evenly distributed in chitosan polymerization network. (2) CH-pectin type [21]. The formation of CH-pectin complex is mainly through two interactions. The first one is hydrogen bonding. It is established because -OH groups produce from pectin molecules connected with -NH_2_, -OH or N-acetyl groups on chitosan molecules. Secondly, stronger links are formed through electrostatic interaction. In an acetic acid solution, -NH_2_ on chitosan molecule is partially protonated into NH_3_^+^, which produce strong ionic bond by combining with free COO^−^ group produced from pectin molecules in aqueous solution. (3) CH-Alginate type [22] Except electrostatic interaction and hydrogen bonding, a typical feature in this type is the existence of cation (e.g., Ca^2+^) crosslinking. It forms a stable complex structure with chitosan molecules. Thus, the formed film shows good mechanical and barrier properties.

In this study, *Zanthoxylum* extracts as an additive agent includes relatively complex components in it. As a results, diverse types of interactions would be established when *Zanthoxylum* extracts mixed with chitosan molecules (as shown in Figure 1C). Generally, the whole mechanical properties of a composite material always depend on its weakest link, while WVP is conversely dictated by the most active composite. It can explain why the introduction of *Zanthoxylum* extract decreased the mechanical properties of the composite film and increased its WVP in this work [4].

### 3.2. Characterization of the Chitosan Films

After a GC-MS analysis, over 99% of components in the *Zanthoxylum* extracts were identified. Main bio-active components in the extracts were listed in Table 1.

**Figure 2:**
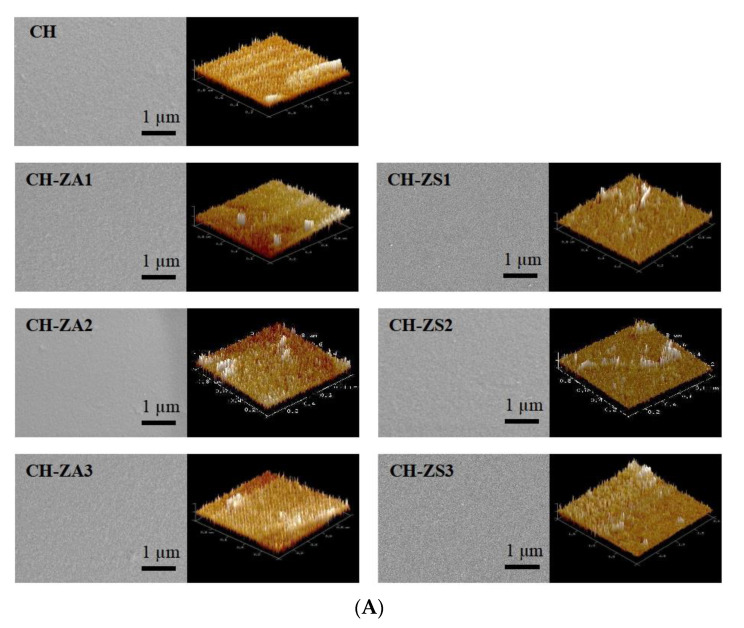

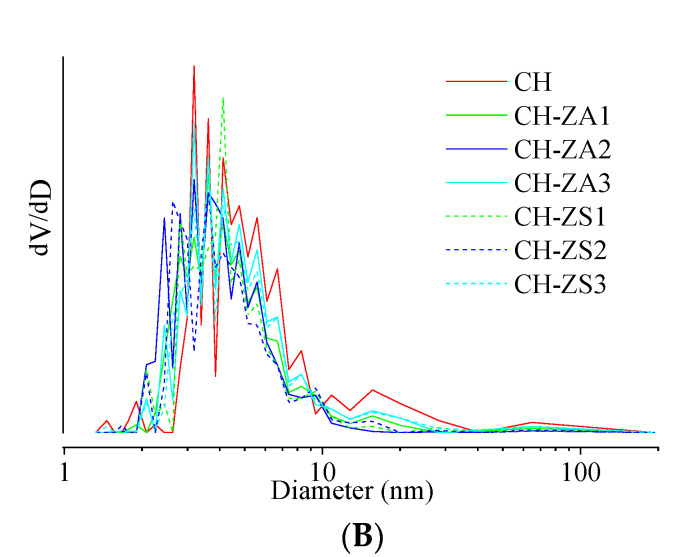
Physical and mechanical properties of the chitosan films. (**A**) SEM and AFM morphology; and (**B**) Porous properties.

Figure 2A displayed the micro-structure of the chitosan films. As it shown, it could not be found obvious difference among different films, and each prepared film had a homogeneous interface, indicating some stable structure may be established between chitosan and *Zanthoxylum* compositions. Meanwhile, as exhibited in Table 2, significant difference in color parameters could be found among different films. Possible reason was that *Zanthoxylum* extracts may results in some changes in absorbance of the chitosan films.

As listed in Table 3, the tensile strength of the films decreased slightly after the addition of the *Zanthoxylum* extracts. The reason can be attributed to that homogeneity and continuity of a composite material strongly affected its mechanical properties, while the introduction of *Zanthoxylum* extracts brings many immiscible components in the films [37]. In addition, Table 3 demonstrated the inhibition of the *Zanthoxylum* extracts on the water vapor permeability of the films, indicating the weak hydrophilicity of *Zanthoxylum* extracts, as the water vapor permeability of a film was greatly dependent on its hydrophilicity [38].

Porosity is also an important property for a film. As shown in Figure 2B, major responses of pure chitosan were around the diameter of 2.8~5.6 nm. The introduction of *Zanthoxylum* extracts in chitosan broadens the peaks around the range of 2.1~8.3 nm, due to phenolic and olefin compounds in the *Zanthoxylum* extracts can results in more ionization in chitosan. 

### 3.3. Antioxidant and Antimicrobial Activity of the Chitosan Films

The antioxidant activity of pure chitosan was revealed in Table 4, represented by the DPPH inhibition rate of 5.27% and the ABTS^+^ inhibition rate of 6.24%. A possible mechanism has been reported by Shaheen et al. [39], through free iron ion. The free iron ions can form a complex with chitosan by chelation. It inhibits the catalytic activity of iron ions in the oxidation reaction.

Both ZA extract and ZS extract exhibited remarkable antioxidant activity. The DPPH IC_50_ of ZA extracts and ZS extracts were 22.37 µg/mL and 24.16 µg/mL, respectively, while the corresponding ABTS^+^ IC_50_ were 15.98 µg/mL and 17.47 µg/mL, respectively. These activities were a little weaker than BHT (DPPH IC_50_ is 19.42 µg/mL and ABTS^+^ IC_50_ is 11.70 µg/mL) and ascorbic acid (DPPH IC_50_ is 17.14 µg/mL and ABTS^+^ IC_50_ is 10.08 µg/mL) [4], while they were higher than many other extracts [40]. The antioxidant activity of *Zanthoxylum* extracts is originated from two possible mechanism. The first one is that some compositions in *Zanthoxylum* extracts act as scavenger of free radicals [41], especially someone has abundant double bond. The other one works by suppressing the enzymes that can promote oxidant effects [42]. As a result, the introduction of *Zanthoxylum* extracts significantly enhanced the antioxidant activity of the chitosan films.

Table 5 exhibited the results of antimicrobial assay. As it shown, the antimicrobial activity of ZA and ZS extracts were similar, represented by the inhibition zones for three microbial species ranged from 16.6 mm to 34.8 mm with varying concentrations. The antimicrobial activity of *Zanthoxylum* extracts is mainly resulted from phenolic compounds in the *Zanthoxylum* extracts, which can result in alteration of lipoprotein membranes (e.g., glucose as carriers to passively transport small hydrophilic molecules), causing impairment of cellular ionic homeostasis, changes of pH in the cell, and destruction of cellular integrity [43,44].

The enhancement of *Zanthoxylum* extracts on the antimicrobial activity of chitosan films could be explained using Lewis acid-base theory [45]. Dissolving *Zanthoxylum* extracts in water produces H^+^ (Thereby *Zanthoxylum* extracts could be considered as a week acid) [46]. It can act as acceptor of electrons and enhance positive charge density on the surface of chitosan. The modified chitosan molecules become easier to adsorb and destroy the cell membrane of bacteria with negatively charges [47].

### 3.4. Sensory and Physical Properties of the Preserved Apples during the Storage

**Figure 3:**
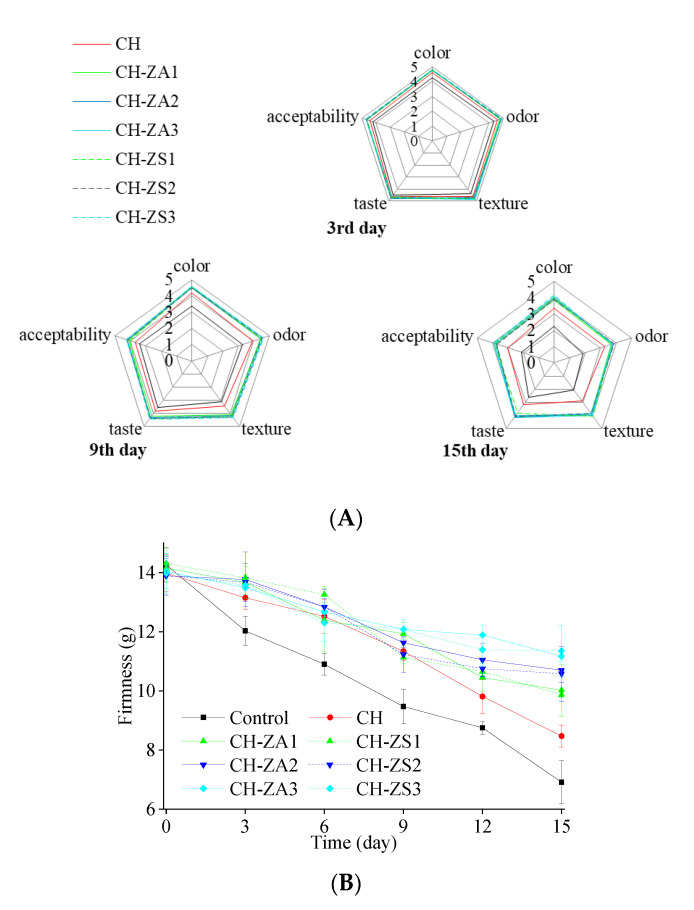

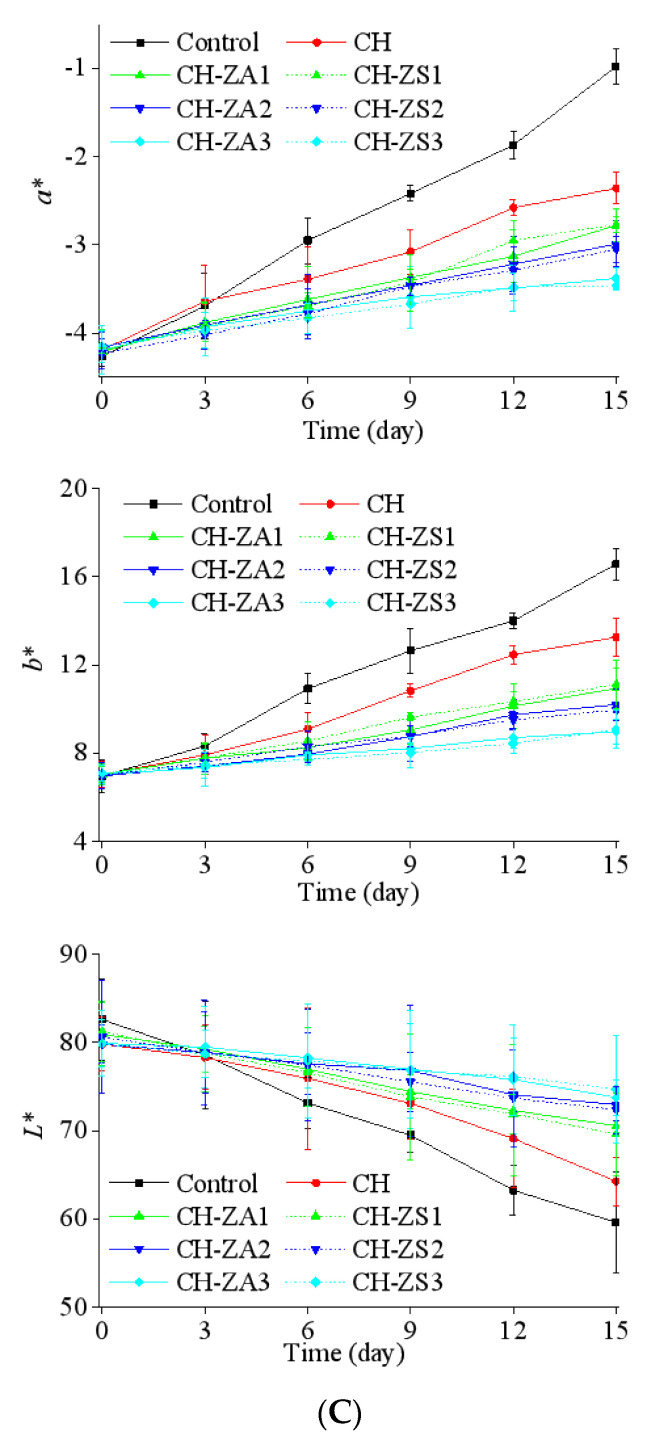
Sensory and physical properties of the preserved apples. (**A**) Sensory scores; (**B**) Firmness; and (**C**) Surface color properties.

Generally, the differences in sensory properties of fruits are affected by species, maturity and size, as well as culture circumstance, harvesting season and storage atmosphere. In this study, sensory score showed decreasing trends from initial score of 5 (as Figure 3A). Control samples had most significant declines in sensory scores compared to the other samples, which reached 1.86~2.64 at the end of the storage. By comparison, the scores of the samples with CH shell were still kept at 2.92~3.36, and CH-extracts shell were still more than 3.7. The phenomenon can be attributed to that the inhibition of chitosan shell on spoilage process of the preserved products. The effects can be enhanced by the introduction of *Zanthoxylum* extracts.

When it comes to firmness, decreasing trends were exhibited in both control and glazed samples (As shown in Figure 3B). In the control samples, such declines were more significant, compared to the samples with CH shells. As a climacteric fruit species, apple softening after harvesting is mainly caused by enzymatic hydrolysis that destroy the cell walls [30]. The introduction of *Zanthoxylum* extracts would result in a acidic environment, in which the activity of the enzymatic hydrolysis was inhibited to a great extent.

Color is a visible index to evaluate the quality of food [17]. For fresh-cut apple, browning is a main external problem that significantly affects the marketability [4]. In this work, obvious browning can be found in most of control samples at the end of the storage, which can be represented by the variations in color parameters. As shown in Figure 3C, the obvious increase of a* and b* revealed that the sample turned from green to red and from blue to yellow, while the drop of L meant that the sample darkened. The combined effect of these variations is sample browning. Meanwhile, almost no browning obviously occurred in the samples with shells, which reflected in the color parameters was less variations during the storage.

### 3.5. Microbial Counts of the Preserved Apples during the Storage

**Figure 4:**
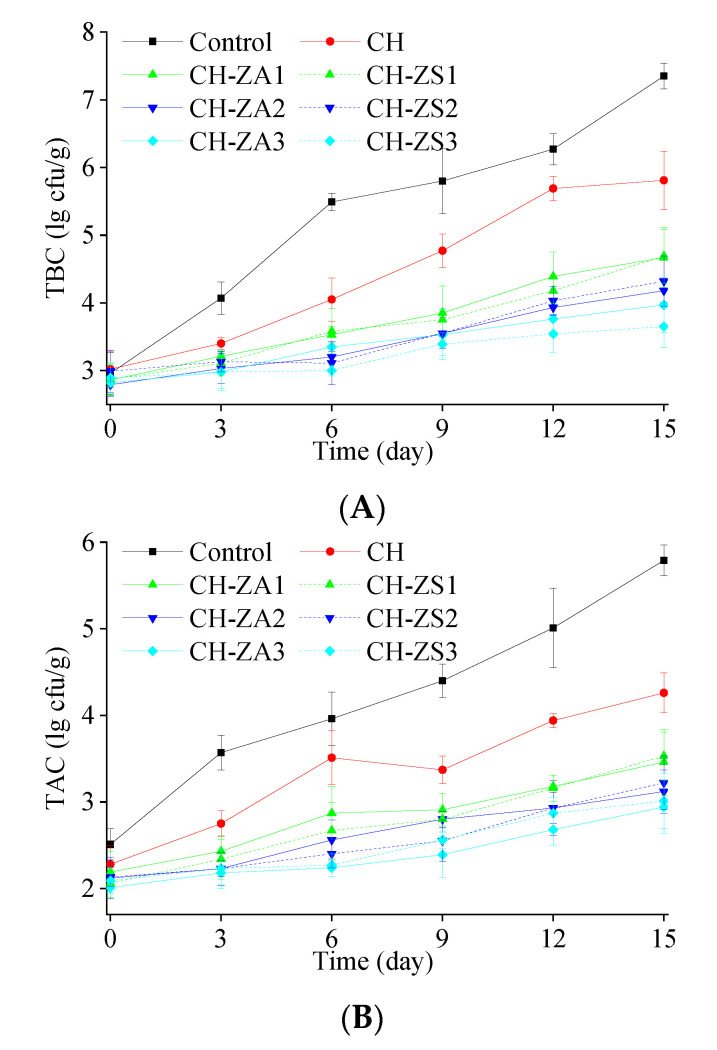
Microbial counts of the preserved apple samples during the storage. (**A**) TBC; and (**B**) TAC.

As remarkable antimicrobial agents, *Zanthoxylum* extracts displayed good antimicrobial effects as a additive agents in chitosan. As shown in Figure 4, upstream trends of microbial counts were presented in both control and treated groups, and such growths were obviously suppressed by the shells. At the beginning of the storage, the initial TBC in all samples were ~2.9 log CFU/g. In the end, compared to the final counts of 7.35 log CFU/g in the control samples and 5.81 log CFU/g in the samples with CH shell, the final TBC were 3.97~4.67 log CFU/g and 3.65~4.69 log CFU/g in the samples with CH-ZA shell and CH-ZS shell, respectively. Meanwhile, TAC exhibited similar trend during the storage. The initial counts in all samples were ~2.2 log CFU/g. After 15 days of storage, the counts increase to 5.97 log CFU/g and 4.26 log CFU/g in the control samples and CH samples, while they were 2.94~3.46 log CFU/g in the samples with CH-ZA shell and 3.01~3.53 log CFU/g in the samples with CH-ZS shell.

### 3.6. Chemical Properties of the Preserved Apples during the Storage

**Figure 5:**
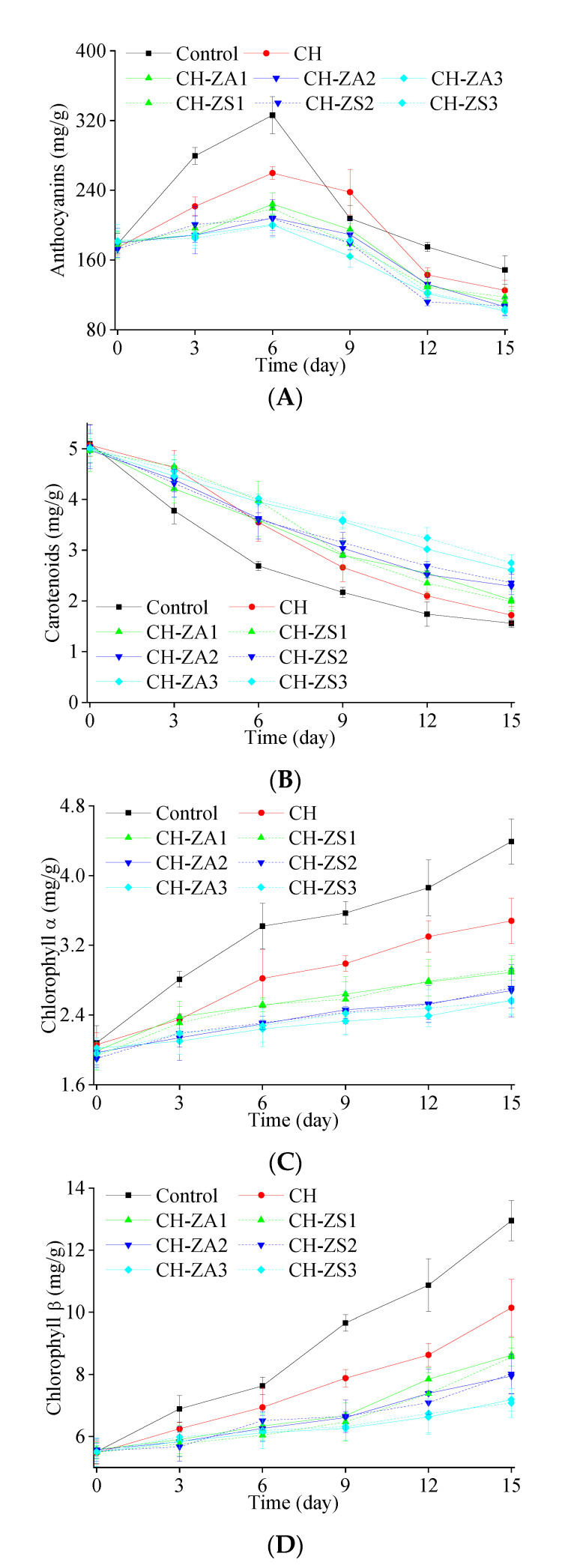
Chemical properties of the preserved apple samples during the storage. (**A**) Anthocyanins; (**B**) Carotenoids; (**C**) Chlorophyll α; and (**D**) Chlorophyll β.

Initially, anthocyanins content in the samples was ~180 mg/kg (as Figure 5A). At the early stage of the storage, anthocyanins content showed increasing trends, then the trends were went downstream at the second half of the storage. The increase can be due to the transformation from phenylalanine through complex synthetic pathway during the process of apple ripening, while the declines can result from more anthocyanins content decomposed through enzymatic reaction. Obviously, such variations of the preserved apples were suppressed by all the bio-polymer shells.

When it came to the carotenoids content, the initial content for both control and treated samples was ~5.0 mg/g. Subsequently, it showed decreasing trends throughout all the storage process (as Figure 5B). Compared to the samples with extracts modified shell, carotenoids contents in the control apples showed more obvious decrease. The reason is that *Zanthoxylum* extracts retard the decomposition process of carotenoids content in the preserved samples during the storage.

Figure 5C,D displayed the increase of both chlorophyll α and chlorophyll during the storage. In the control samples, highest contents were obtained in both chlorophyll α and chlorophyll β, represented by the values varied from 2.08 mg/g to 4.39 mg/g and from 5.51 mg/g to 12.95 mg/g during the storage, respectively. The followed contents were detected in the samples with CH shell. The initial contents were 2.06 mg/g and 5.47 mg/g and increased to 3.48 mg/g and 10.41 mg/g at the end of the storage, respectively. The lowest chlorophyll content was found in the samples with modified shells. During the storage, the chlorophyll content in the samples with CH-ZA shell increased from 2.02 mg/g (chlorophyll α) and 5.49 mg/g (chlorophyll β) to 2.57 mg/g and 7.19 mg/g, while the contents in the samples with CH-ZS shell increased from 1.96 mg/g (chlorophyll α) and 5.50 mg/g (chlorophyll β) to 2.56 mg/g and 7.08 mg/g. The reason for the trends is that CH shell as a protective barrier can effectively protect the preserved products from the contact of oxygen, which may help to accelerate breakdown process of cells and release the chlorophyll contents [4].

## 4. Conclusions

Twelve kinds of identified active components in extracts from ZA and ZS consisted of over 50% of total. The ZA or ZS extracts were used as additive agents to modify chitosan film. Results revealed that the modified material showed some variations in micro-appearances, color parameters, mechanical strength, water vapor permeability. Moreover, the modified material was endowed with more significant antioxidant and antimicrobial activity. On this basis, the modified material was glazed as surface shell to preserve fresh-cut apple. It can be found that the decline process of postharvest quality of the preserved apple samples were suppressed. The findings in this study revealed promising potential of the glazing shell in food application.

## Figures and Tables

**Table 1:** Bioactivity of main composition in the *Zanthoxylum* extracts.

RI ^1^	Components	% in ZA ^2^	% In ZS ^2^	Reported Bioactivity
863	*trans*-2-Hexenal	3.33	3.59	Antimicrobial, antioxidant [23]
1030	Limonene	2.96	7.40	Antimicrobial, antioxidant [24]
1036	Eucalyptol	13.31	-	Antimicrobial [25]
1078	*cis*-Linalool oxide	4.97	1.33	Antimicrobial [26]
1099	Linalool	4.86	3.92	Antimicrobial [27], antioxidant [28]
1168	Borneol	0.23	23.39	Antimicrobial, antioxidant [29]
1229	Estragole	12.19	0.17	Antimicrobial [30]
1357	Eugenol	1.74	2.94	Antimicrobial, antioxidant [31]
1390	β-Elemene	0.59	12.48	Antioxidant [32]
1418	β-Caryophyllene	6.77	0.11	Antimicrobial [33], antioxidant [34]
1486	Germacrene D	4.10	4.92	Antimicrobial [35]
1578	Caryophyllene oxide	1.72	4.70	Antimicrobial [36]
	Total	56.77	64.95	

^1^ Retention index relative to n-alkanes on HP-5 MS capillary column. ^2^ Relative area (peak area relative to the total peak area).

**Table 2:** Color parameters (L*, a*, and b*) of the chitosan films.

Materials	L*	a*	b*
CH	71.33 ± 6.14 a	3.97 ± 0.28 a	−5.26 ± 0.42 g
CH-ZA1	61.97 ± 3.78 b	2.85 ± 0.31 c	−2.04 ± 0.83 f
CH-ZA2	58.21 ± 4.49 c	2.38 ± 0.15 d	2.31 ± 0.20 c
CH-ZA3	58.76 ± 5.22 c	1.86 ± 0.22 f	7.67 ± 0.57 a
CH-ZS1	63.72 ± 4.95 b	3.09 ± 0.27 b	−1.60 ± 0.38 e
CH-ZS2	60.04 ± 7.48 bc	2.10 ± 0.35 e	0.86 ± 0.11 d
CH-ZS3	56.38 ± 3.57 c	0.96 ± 0.15 g	6.75 ± 0.53 b

a–g mean the values followed by different letters differ significantly by LSD test (*p* ≤ 0.05).

**Table 3:** Mechanical properties and permeability of the chitosan films.

Materials	Thickness (µm)	Tensile Strength (MPa)	Water Vapor Permeability (10^−10^ g·m^−1^·h^−1^·Pa^−1^)
CH	62 ± 4 ab	4.8 ± 0.7 a	5.97 ± 0.42 a
CH-ZA1	65 ± 3 a	4.5 ± 0.5 b	5.38 ± 0.36 b
CH-ZA2	63 ± 5 ab	4.1 ± 0.6 cd	5.04 ± 0.42 cd
CH-ZA3	58 ± 7 c	4.3 ± 0.3 c	5.15 ± 0.51 c
CH-ZS1	62 ± 6 ab	4.0 ± 0.4 d	5.27 ± 0.18 bc
CH-ZS2	57 ± 5 c	4.2 ± 0.3 c	4.96 ± 0.47 cd
CH-ZS3	61 ± 2 b	4.2 ± 0.8 c	4.89 ± 0.36 d

a–d mean the values followed by different letters differ significantly by LSD test (*p* ≤ 0.05).

**Table 4:** Antioxidant activity of the materials.

Materials		DPPH		ABTS^+^	
		Inhibition (%)	IC50 (µg/mL)	Inhibition (%)	IC50 (µg/mL)
Films	CH	5.27 ± 1.02 f	-	6.24 ± 0.82 e	-
	CH-ZA1	30.95 ± 4.27 d	-	32.85 ± 2.67 d	-
	CH-ZA2	35.68 ± 2.99 b	-	38.64 ± 5.31 b	
	CH-ZA3	38.37 ± 5.74 a	-	41.57 ± 4.83 a	
	CH-ZS1	28.64 ± 1.58 e	-	35.46 ± 5.92 c	-
	CH-ZS2	32.90 ± 4.69 c	-	36.75 ± 2.64 c	-
	CH-ZS3	37.38 ± 2.97 a	-	40.88 ± 4.27 a	-
ZA extracts	5 µg/mL	38.01 ± 6.32 e	22.37	40.27 ± 5.23 g	15.98
	10 µg/mL	42.63 ± 2.80 d		44.32 ± 6.90 f	
	20 µg/mL	48.27 ± 5.37 c		51.09 ± 3.68 e	
	40 µg/mL	53.60 ± 9.44 b		58.14 ± 4.39 d	
	60 µg/mL	60.22 ± 4.88 a		64.02 ± 8.16 c	
ZS extracts	5 µg/mL	35.90 ± 5.25 f	24.16	33.92 ± 4.46 h	17.47
	10 µg/mL	40.78 ± 2.46 e		42.01 ± 2.65 gf	
	20 µg/mL	48.91 ± 6.53 c		54.57 ± 6.83 e	
	40 µg/mL	55.14 ± 2.88 b		68.64 ± 5.79 b	
	60 µg/mL	58.02 ± 6.96 ab		78.90 ± 8.63 a	

a–h mean the values followed by different letters differ significantly by LSD test (*p* ≤ 0.05).

**Table 5:** Antimicrobial activity of the chitosan films against different microbial species.

Materials		Inhibition Zone (mm)		
		*S. aureus*	*P. aeroginosa*	*E. coli*
Films	CH	2.9 ± 0.7 e	1.7 ± 0.6 g	2.1 ± 1.0 g
	CH-ZA1	11.2 ± 2.0 d	9.6 ± 1.8 f	10.1 ± 2.6 e
	CH-ZA2	12.6 ± 3.3 c	10.9 ± 2.7 d	10.6 ± 2.7 d
	CH-ZA3	15.2 ± 2.8 b	12.2 ± 2.2 c	12.4 ± 1.3 b
	CH-ZS1	11.6 ± 1.1 d	10.3 ± 1.6 e	9.7 ± 2.7 f
	CH-ZS2	14.6 ± 1.8 b	12.8 ± 3.6 b	11.5 ± 0.9 c
	CH-ZS3	16.3 ± 3.5 a	13.9 ± 3.8 a	14.6 ± 4.1 a
ZA extracts	10 μL	24.6 ± 2.0 f	18.0 ± 0.4 e	16.6 ± 1.2 e
	20 μL	29.5 ± 3.8 d	19.5 ± 2.9 d	19.6 ± 4.7 c
	30 μL	32.6 ± 4.5 b	25.3 ± 3.3 a	22.1 ± 3.2 b
ZS extracts	10 μL	26.4 ± 1.2 e	16.3 ± 1.2 f	17.6 ± 3.1 d
	20 μL	31.5 ± 1.3 c	20.8 ± 3.4 c	19.9 ± 2.3 c
	30 μL	34.8 ± 2.9 a	23.5 ± 2.6 b	24.7 ± 1.6 a
Antibiotic	Penicillin	45.5 ± 0.7	39.1 ± 7.0	9.0 ± 1.0
	Streptomycin	38.5± 0.5	20.3 ± 0.6	-
	Ampicillin	21.0 ± 0.0	-	12.0 ± 1.0

a–g mean the values followed by different letters differ significantly by LSD test (*p* ≤ 0.05).

## Data Availability

Not applicable.

## References

[1] Realini C.E., Marcos B. (2014). Active and intelligent packaging systems for a modern society. Meat Sci..

[2] Kumarihami H.M.P.C., Kim Y.H., Kwack Y.B., Kim J., Kim J.G. (2022). Application of chitosan as edible coating to enhance storability and fruit quality of Kiwifruit: A review. Sci. Hortic..

[3] Vilela C., Pinto R.J.B., Coelho J., Domingues M.R.M., Daina S., Sadocco P., Santos S.A.O., Freire C.S.R. (2017). Bioactive chitosan/ellagic acid films with UV-light protection for active food packaging. Food Hydrocoll..

[4] Wang W.X., Zhang Y.L., Yang Z., He Q. (2021). Effects of incorporation with clove (*Eugenia caryophyllata*) essential oil (CEO) on overall performance of chitosan as active coating. Int. J. Biol. Macromol..

[5] He Q., Gong B., He J.P., Yang X.C., Xiao K.J., Zhu L. (2018). A novel superchilling storage-ice glazing (SS-IG) approach using biopolymer-based composite hydrogel to delay microbiological spoilage and organic oxidation of preserved tilapia. J. Sci. Food Agric..

[6] Rubilar J.F., Cruz R.M., Silva H.D., Vicente A.A., Khmelinskii I., Vieira M.C. (2013). Physico-mechanical properties of chitosan films with carvacrol and grape seed extract. J. Food Eng..

[7] Kerch G. (2015). Chitosan films and coatings prevent losses of fresh fruit nutritional quality: A review. Trends Food Sci. Technol..

[8] Hosseini S.F., Zandi M., Rezaei M., Farahmandghavi F. (2013). Two-step method for encapsulation of oregano essential oil in chitosan nanoparticles: Preparation, characterization and in vitro release study. Carbohyd. Polym..

[9] Atares L., Chiralt A. (2016). Essential oils as additives in biodegradable films and coatings for active food packaging. Trends Food Sci. Technol..

[10] He Q., Wang W.X., Zhu L. (2018). Larvicidal activity of *Zanthoxylum acanthopodium* essential oil against the malaria mosquitoes, *Anopheles anthropophagus* and *Anopheles sinensis*. Malar. J..

[11] Qi H., Wang W.X., Dai J.L., Zhu L. (2015). In vitro anthelmintic activity of *Zanthoxylum*
*simulans* essential oil against *Haemonchus contortus*. Vet. Parasitol..

[12] He Q., Shen Y., Xiao K.J., Xi J.Y., Qiu X.P. (2016). Alcohol electro-oxidation on platinumeceria/graphene nanosheet in alkaline solutions. Int. J. Hydrogen Energy.

[13] He Q., Xiao K.J. (2016). The effects of tangerine peel (*Citri reticulatae pericarpium*) essential oils as glazing layer on freshness preservation of bream (*Megalobrama amblycephala*) during superchilling storage. Food Control.

[14] Liu J., Liu S., Wu Q.Q., Gu Y.Y., Kan J., Jin C.H. (2017). Effect of protocatechuic acid incorporation on the physical, mechanical, structural and antioxidant properties of chitosan film. Food Hydrocoll..

[15] He Q., Gong B., He J.P., Xiao K.J. (2019). A novel superchilling storage-ice glazing (SS-IG) approach using antioxidative and antimicrobial essential oil (EO) for freshness-keeping of sea bass (*Dicentrarchus labrax*). Aquaculture.

[16] He Q., Zhu L., Shen Y., Lin X.D., Xiao K.J. (2015). Evaluation of the effects of frozen storage on the microstructure of tilapia (*Perciformes: Cichlidae*) through fractal method. LWT-Food Sci. Technol..

[17] He Q., Xiao K.J. (2018). Quality of broccoli (*Brassica oleracea L. var. italica*) in modified atmosphere packaging made by gas barrier-gas promoter blending materials. Postharvest Biol. Technol..

[18] Zhang D., Quantick P.C. (1997). Effects of chitosan coating on enzymatic browning and decay during postharvest storage of litchi (*Litchi chinensis Sonn.*) fruit. Postharvest Biol. Technol..

[19] Li Y., Yang X.Y., Wang X.Y., Zhao M.N., Feng J., Xia X.F. (2021). Research progress on the film-forming mechanism and characteristics of chitosan-based composite membranes. Sci. Technol. Food Ind..

[20] Sindhu M., Brahmakumarbt M., Emilia T.A. (2006). Microstructural imaging and characterization of the mechanical, chemical, thermal, and swelling properties of starch-chitosan blend films. Biopolymers.

[21] Heba G.R., Zhao G.H. (2019). Physicochemical properties of the edible films from the blends of high methoxyl apple pectin and chitosan. Int. J. Biol. Macromol..

[22] Yan X.L., Khor E., Lim L.Y. (2001). Chitosan-alginate films prepared with chitosans of different molecular weights. J. Biomed. Mater. Res..

[23] Lu H.B., Xu S.Y., Zhang W.J., Xu C.M., Li B.X., Zhang D.X., Mu W., Liu F. (2017). Nematicidal activity of trans-2-Hexenal against southern Root-Knot nematode (*Meloidogyne incognita*) on tomato plants. J. Agric. Food Chem..

[24] Hafsa J., Smach M.A., Khedher M.R.B., Charfeddine B., Limem K., Majdoub H., Rouatbi H. (2016). Physical, antioxidant and antimicrobial properties of chitosan films containing Eucalyptus globulus essential oil. LWT-Food Sci. Technol..

[25] Morcia C., Malnati M., Terzi V. (2012). In vitro antifungal activity of terpinen-4-ol, eugenol, carvone, 1, 8-cineole (eucalyptol) and thymol against mycotoxigenic plant pathogens. Food Addit. Contam. A.

[26] Behnaz T., Mehdi R., Ahmad A. (2017). Essential oil composition, total phenolic, flavonoid contents, and antioxidant activity of Thymus species collected from different regions of Iran. Food Chem..

[27] Herman A., Tambor K., Herman A. (2016). Linalool affects the antimicrobial efficacy of essential oils. Curr. Microbiol..

[28] Seol G.H., Kang P., Lee H.S., Seol G.H. (2016). Antioxidant activity of linalool in patients with carpal tunnel syndrome. BMC Neurol..

[29] Horvathova E., Navarova J., Galova E., Sevcovicova A., Chodakova L., Snahnicanova Z., Melusova M., Kozics K., Slamenova D. (2014). Assessment of antioxidative, chelating, and DNA-protective effects of selected essential oil components (eugenol, carvacrol, thymol, borneol, eucalyptol) of plants and intact rosmarinus officinalis oil. J. Agric. Food Chem..

[30] Andrade T.C.B., de Lima S.G., Freitas R.M., Rocha M.S., Islam T., da Silva T.G., Militao G.C.G. (2015). Isolation, characterization and evaluation of antimicrobial and cytotoxic activity of estragole, obtained from the essential oil of croton zehntneri (*euphorbiaceae*). An. Acad. Bras. Cienc..

[31] Lee J.Y., Jung M.Y. (2017). Effects and mechanisms of eugenol, isoeugenol, coniferylaldehyde and dihydroeugenol on the riboflavin-sensitized photooxidation of alpha-terpinene in methanol. Food Chem..

[32] Chen J.C., Duan W.L., Bai R.R., Yao H.Q., Wu X.M., Shang J., Xu J.Y. (2014). Design, synthesis and antioxidant activity evaluation of novel beta-elemene derivatives. Bioorg. Med. Chem. Lett..

[33] Chang H.J., Kim J.M., Lee J.C., Kim W.K., Chun H.S. (2013). Protective effect of beta-caryophyllene, a natural bicyclic sesquiterpene, against cerebral ischemic injury. J. Med. Food.

[34] Joshi R.K. (2016). Leucas aspera (willd) link essential oil from India: Beta-caryophyllene and 1-octen-3-ol chemotypes. J. Chromat. Sci..

[35] Setzer W.N. (2018). Germacrene D cyclization: An Ab initio investigation. Int. J. Mol. Sci..

[36] Gyrdymova Y.V., Izmestev E.S., Rubtsova S.A., Kutchin A.V. (2016). Synthesis and oxidation of sulfides based on caryophyllene oxide and phenylmethanethiol. Russ. J. Org. Chem..

[37] Chatkitanan T., Harnkarnsujarit N. (2021). Effects of nitrite incorporated active films on quality of pork. Meat Sci..

[38] Wangprasertkul J., Siriwattanapong R., Harnkarnsujarit N. (2021). Antifungal packaging of sorbate and benzoate incorporated biodegradable films for fresh noodles. Food Control.

[39] Shaheen M.S., Shaaban H.A., Hussein A.M.S., Ahmed M.B.M., El-Massry K., El-Ghorab A. (2016). Evaluation of chitosan/fructose model as an antioxidant and antimicrobial agent for shelf life extension of beef meat during freezing. Pol. J. Food Nutr. Sci..

[40] He Q., Li Z.Y., Yang Z., Zhang Y.C., Liu J. (2020). A superchilling storage–ice glazing (SS-IG) of Atlantic salmon (*Salmo salar*) sashimi fillets using coating protective layers of Zanthoxylum essential oils (EOs). Aquaculture.

[41] Jing P., Zhao S.J., Jian W.J., Qian B.J., Dong Y., Pang J. (2012). Quantitative studies on structure-DPPH scavenging activity relationships of food phenolic acids. Molecules.

[42] Kundu A., Saha S., Walia S., Ahluwalia V., Kaur C. (2013). Antioxidant potential of essential oil and cadinene sesquiterpenes of *Eupatorium Adenophorum*. Toxicol. Environ. Chem..

[43] Ozek T., Tabanca N., Demirci F., Wedge D.E., Baser K.H.C. (2010). Enantiomeric distribution of some linalool containing essential oils and their biological activities. Rec. Nat. Prod..

[44] Klinmalai P., Srisa A., Laorenza Y., Katekhong W., Harnkarnsujarit N. (2021). Antifungal and plasticization effects of carvacrol in biodegradable poly (lactic acid) and poly (butylene adipate terephthalate) blend films for bakery packaging. LWT-Food Sci. Technol..

[45] Basolo F., Pearson R.G. (1967). Mechanisms of Inorganic Reactions.

[46] Park S.Y., Son B.G., Park Y.H., Kim C.M., Park G., Choi Y.W. (2014). The neuroprotective effects of alpha-iso-cubebene on dopaminergic cell death: Involvement of CREB/Nrf2 signaling. Neurochem. Res..

[47] Valero D., Díaz-Mulaa H.M., Zapataa P.J., Guillen F., Martínez-Romero D., Castillo S., Serrano M. (2013). Effects of alginate edible coating on preserving fruit quality in four plum cultivars during postharvest storage. Postharvest Biol. Technol..

